# Arboviral Diseases in Livestock

**DOI:** 10.3390/v18060672

**Published:** 2026-06-15

**Authors:** Estelle Venter, Antoinette van Schalkwyk

**Affiliations:** 1 Veterinary Science, College of Science and Engineering, James Cook University, Townsville 4811, QLD, Australia; 2 Department of Veterinary Tropical Diseases, Faculty of Veterinary Science, University of Pretoria, Pretoria 0110, South Africa; 3 Agricultural Research Council—Onderstepoort Veterinary Institute, Pretoria 0110, South Africa; vanschalkwyka1@arc.agric.za

## 1. Introduction

In 1984, the then Director of the National Institute of Allergy and Infectious Diseases (NIAID) suggested that HIV was the only emerging infectious disease likely to pose a significant future threat. Similar sentiments had been expressed a decade earlier by Nobel Prize-winning virologist Sir F. Macfarlane Burnet and his colleague Dr. David O. White, who wrote that “to write about infectious disease is almost to write of something that has passed into history…the most likely forecast about the future of infectious disease is that it will be very dull” [1].

Subsequent decades proved these predictions to be remarkably inaccurate. By 2017, the global public health landscape had been transformed by the emergence and reemergence of numerous infectious diseases, culminating in the COVID-19 pandemic in 2020 [2]. Among the pathogens contributing to this changing landscape, arboviruses and other vector-borne viruses have played a particularly important role. Most emerging viruses are zoonotic, infecting both animals and humans, with arthropod vectors often facilitating transmission across species barriers. It is estimated that approximately 75% of emerging viral diseases originate from animal hosts.

Multiple interconnected factors drive the emergence and spread of viral diseases. The movement of animals into new geographic regions, whether through trade, agricultural activities, or natural migration, can introduce pathogens into previously unaffected populations. Between 2000 and 2004 alone, more than one billion animals from 163 countries were legally imported into the United States, despite limited screening for arboviruses [3]. Simultaneously, climate change has altered the distribution and abundance of arthropod vectors, increasing the frequency, geographic range, and impact of transboundary epizootics. The consequences of these diseases extend far beyond animal health, affecting livestock productivity, international trade, food production systems, and global food security.

## 2. Contributions

The five articles featured in this Special Issue highlight the diversity, emergence, detection, and epidemiological significance of livestock-associated viral diseases across multiple regions of the world. Collectively, they illustrate the continuing challenges posed by both established and emerging pathogens and underscore the importance of surveillance, diagnostics, and molecular epidemiology in disease control.

Chernyshev et al. [4]. describe a unique nucleotide polymorphism in African swine fever virus circulating in East Asia and Central Russia, providing new insights into the molecular evolution and geographic spread of this economically devastating pathogen. Golender et al. [5]. report the development of novel probe-based real-time RT-qPCR assays capable of detecting all currently known strains of bovine ephemeral fever virus, representing an important advancement in diagnostic preparedness and outbreak response.

Delooz et al. [6]. document cattle abortions and congenital malformations associated with bluetongue virus serotype 3 in southern Belgium during 2024, highlighting the significant animal health and production consequences of newly emerging bluetongue virus strains in Europe. Using unbiased sequencing approaches, Homonnay et al. [7]. identify Batai virus as an emerging orthobunyavirus in Hungarian cattle, demonstrating the value of next-generation sequencing technologies for the detection of previously overlooked viral pathogens. Finally, Adamu et al. [8]. present serological evidence of exposure to Akabane virus, bluetongue virus, and bovine ephemeral fever virus in feral water buffalo populations in northern Australia, emphasizing the potential role of wildlife reservoirs in the maintenance and transmission of livestock pathogens.

Together, these contributions provide valuable perspectives on viral emergence, pathogen evolution, diagnostic innovation, and disease ecology. They reinforce the need for integrated One Health approaches that recognize the interconnectedness of animal, human, and environmental health in addressing the challenges posed by emerging and reemerging viral diseases.
